# Allergen Avoidance in Allergic Asthma

**DOI:** 10.3389/fped.2017.00103

**Published:** 2017-05-10

**Authors:** Francesca Cipriani, Elisabetta Calamelli, Giampaolo Ricci

**Affiliations:** ^1^Pediatric Unit, S. Orsola-Malpighi Hospital, Department of Medical and Surgical Sciences, University of Bologna, Bologna, Italy; ^2^Pediatric and Neonatology Unit, Imola Hospital, Bologna, Italy

**Keywords:** allergen, asthma, avoidance, exposure, childhood, mite, mold, pets

## Abstract

Allergic asthma is the most frequent disease among the chronic respiratory disorders in pediatric age with an important social impact. In the last years, many efforts have been made to identify effective preventive approaches to get a better control of symptoms and to obtain the best future outcomes for the patients. In patients with allergic asthma triggered by the exposure to indoor allergens, the avoidance is the first intervention to prevent the appearance or the worsening of bronchial symptoms. This review article summarized the most recent evidence from literature about the efficacy of specific control interventions for the most important allergens. Even if a wide spectrum of interventions has been suggested and may help to reduce exposure to trigger allergy for sensitized patients suffering from respiratory allergy, evidence supporting the efficacy of these approaches is still weak and subject of controversy. However, the exposure control to specific airborne allergens is still widely recommended and may be effective as part of a holistic approach to reduce the severity of allergic respiratory symptoms in sensitized individuals.

## Introduction

Allergic asthma is a chronic respiratory disease that affects millions of people worldwide with a significant burden to patients and the community; its prevalence varies among countries from 1 to 18% (1, 2). Over the past decades, a great interest has been focused on recognizing effective treatments and preventive approaches to reduce the burden of asthma, especially early in life and in the pediatric population. Current research are in fact directed to understand the factors related to the development and worsening of asthma and to find new strategies to reduce exacerbations, its epidemiological and socioeconomic impacts, and to improve the quality of life.

Asthma often occurs in patients sensitized to indoor allergens (e.g., mites and pets). Data from German MAS birth cohort study showed that schoolchildren sensitized to perennial allergens with high exposure early in life are more prone to develop an impaired lung function at school age than children without sensitization or sensitized to indoor allergens but with a low exposure in the first years of life (3). This study highlighted the importance of avoidance of allergen source as a fundamental intervention in the management of allergic patient.

In patients with allergic asthma triggered by the exposure to particular allergens, the avoidance is the first measure to prevent the appearance or the worsening of bronchial symptoms.

Therefore, since a key factor for the development of respiratory allergy is the exposure to airborne allergens, many avoidance strategies have carried out to reduce allergens exposure to avoid the elicitation of symptoms.

The aim of this review article is to focus on the most effective interventions for reducing exposure to the most common airborne allergens in patients with allergic asthma. Primary prevention of allergic sensitization and allergic asthma, which includes also percutaneous sensitization and sensitization of oral and the gastrointestinal tract falls outside the purpose of this review.

References were identified by searches of PubMed and UpToDate and restricted to English articles. Search terms used were “pediatric asthma,” “allergen avoidance,” “pollen avoidance,” “pets avoidance,” and “molds avoidance.” We included only meta-analysis, randomized controlled trials, reviews, and systematic review articles pertaining to humans.

### The Guilty: Focus on Airborne Allergen Sources

- *House dust mites (HDM)* represent the typical indoor perennial allergens. *Dermatophagoides pteronyssinus* (European HDM), *Dermatophagoides farinae* (American HDM), and *Blomia tropicalis* represent the most important HDM species. The most notable mite allergens are Der p 1 and Der f 1, but recently many other molecules have been characterized and studied in patient sensitized to mite allergens (4). HDM belong to arthropods, in particular to the class of arachnids and their average size is about 0.33 mm. They found sustenance from skin cells that flake off and warm and humid environments facilitate their survival. HDM life can be 6–8 weeks long and females are able to produce 40–80 eggs during their life (5). The life cycle takes ~3–4 weeks. Fecal particles contain a complex mixture of allergenic mite-derived proteins, endotoxin, enzymes, and mite and bacterial DNA, all of which can be immunostimulatory (6, 7). The highest dust mite concentrations are in mattresses (8), but they may be found also in house dust, bedding, upholstered furniture, carpets, and curtains. Their growth and reproduction are facilitated by modern house insulation, higher temperatures, and in particular in presence of high indoor humidity. Dust mite infestation is far less common in arid and high-altitude climates, such as the mountain states and southwestern USA.
- *Pets* (furry animals) are a common source of allergens (9) and sensitization to cats’ and dogs’ main allergens is well known to be associated with severe asthma in childhood (10, 11). The major cat allergen Fel d 1 (secretoglobulin) is found in cats’ skin and fur mainly as product of sebaceous and salivary glands (9), whereas dog allergens are mainly found in its hair, dander, and saliva, Can f 1 (lipocalin) represents the major allergen (9).
- *Cockroaches*, such as *Blatella germanica* (German cockroach), are able to shed or excreted tiny protein particles with allergenic properties and, therefore, they can act as indoor allergens. *B. germanica* and its derived allergens are particularly important in the cities of North America as highlighted in some case–control studies and provocation studies (12–14). A study of 476 children found that the combination of specific skin test positivity and exposure to cockroach allergen was associated with significantly higher rates of hospitalization, compared to when this combination was absent (0.37 vs 0.11 hospitalizations/child/year) (15).
- *Pests*, such as mice and rats, may represent a source of allergens, by secreting major allergens, such as Mus m1 and Rat 1, in their urine. Rodents’ main allergens represent relevant allergenic source in schools, labs, as well as in the domestic environments (16, 17). Mus m 1 is the major mouse allergen, and it has been detected in mouse dander, urine, and hair (18) and as well as furry animals’ allergens can be present even in homes without mice (19).
- *Molds* represent both indoor (perennial) and outdoor (seasonal) allergens. Indoors, molds can be found in any moist, dark place, while outdoors they results from vegetation degradation. Mold floats easily in the air. The high peak of outdoor spores can be identified in the mid-summer; dry air spores, such as those of *Alternaria* and *Cladosporium*, peak especially in the afternoon hours under low humidity. Mold spores need a relative humidity >65%, a temperature between 50 and 90°F (10–32°C), and organic matter as their nutrient base to grow.

Fungal spores may elicit both seasonal and perennial allergic symptoms. Alternaria is the most prevalent mold in dry, warm climates, and it is commonly found in soil, seeds, and plants. *Cladosporium* is the most commonly identified outdoor fungus and also the most prevalent spore in temperate regions, mainly found in decaying plant material.

*Aspergillus* may be frequently detected in dust, but also in fertilizer masses and vegetation. *Penicillium* may be present in dust, grains, and other foods. All of these molds induce allergic respiratory diseases, such as rhinitis, allergic asthma, and hypersensitivity pneumonia. Evidence showed a link between allergic sensitization and exposure to *Alternaria* and severe asthma (20–23).

- *Pollens’* function in natural mechanisms is to transfer gametes of flowers through current of air or insect spreading (8, 24). Pollens reach the peak concentration in the main countries from spring to summer, while in tropical countries, the pollination period may be longer (8, 24). Mean tree pollens diameter varies from 20 to 50 µm, and single plant can produce up to a million of pollen grains daily. Pollens are released above all in the morning, reaching the peak concentration by the afternoon (25). In Europe, allergic patients with hay fever and/or asthma are often sensitized to grass pollen allergens. A single pollen diameter varies from 10 to 100 µm (26). Most of pollen allergens are removed by the nasal mucosa and upper tracheobronchial tract, while the submicronic pollen-derived bioaerosols (<5 μm) easily reach the lower respiratory tract (27). Microscopic particles (0.5–2.5 µm) named “bioaerosol” are released by pollen ruptures, in example on contact with water and contain major allergens (27, 28).

### Avoidance Measures against Indoor Allergens

An important and widely investigated intervention to obtain a good management of patient with asthma is the control of indoor allergens exposure (29). A wide spectrum of interventions has been suggested and may help to reduce exposure to trigger allergy for sensitized patients suffering from respiratory allergy (Table 1).

**Table 1:** **Interventions to eliminate or reduce airborne allergens in the indoor environment**.

**House dust mite**
Use bed-encasing for mattress and duvet/pillows (pore diameter <10 µm) Wash bedding weekly in hot water and dry in a heated drier Remove dust mite reservoirs (i.e., toys and stuffed animals, carpets) Reduce indoor humidity (less than 50%) Vacuum with a HEPA filter bag Remind that chemicals to kill mites or denature proteins have a modest effect
**Pets**
Remove pet from bedrooms and everywhere the child spends a lot of time Clean accurately upholstered furniture, walls, and carpet Remove upholstered furniture and carpet if possible Encase the mattress and pillows with bed encasing (pore diameter <6 µm) Keep the pet clean with frequent washes Use a HEPA air filter
**Cockroach and pests**
Inspect to detect hiding places debris (grease, kitchen) and identify food sources Store food in sealed containers Exterminate with pesticides or bait traps Remove clutter and seal holes or cracks in the home
**Indoor molds^a^**
Reduce indoor humidity (less than 50%) Remove contaminated carpets, wallpaper, and woodwork Treat washable surfaces with detergent and water and then dry completely Repair water leaks
**Pollens**
Keep windows closed Bath to remove allergens from hair and body Consider to use HEPA air filtration Consider same principles for outdoor mold avoidance

*HEPA, high-efficiency particulate air*.

*^a^Level of evidence is largely based on experts opinion*.

*Modified from Platts-Mills (30) and Baxi and Phipatanakul (8)*.

## House Dust Mites

The rationale of avoidance intervention for HDM is that by reducing or containing the mite population, patient exposure to mite allergens is also reduced, resulting in fewer symptoms. Although an intuitive strategy, avoidance is not supported by robust evidence of efficacy and is still the subject of controversy, mainly because of the impartiality of inclusion criteria in reviews and because the actual contribution of indoor environmental factors is difficult to demonstrate scientifically (31–34). Despite this, avoidance is still widely recommended to reduce the severity of allergic respiratory symptoms in sensitized individuals and may be effective as part of a holistic approach combining avoidance of tobacco smoke, improved education, and regular assessment (35, 36).

### Which Avoidance Measures Should Be Recommended to HDM-Sensitized Children with Asthma?

Neither the mites nor their debris can be seen under normal circumstances, therefore education of patients regarding dust mites avoidance may be difficult.

Control measures should be based on allergen exposure monitoring performed according to well-defined and validated methods. It’s imperative at this purpose to report the presence of the sources of allergens. The choice of optimal procedures to monitoring depends on the setting and allergens’ source. Indoor airborne allergen levels may be assessed in settled dust or in an air sample. Bed, carpet, or sofa is the favorite sources to collect dust sample by using a vacuum cleaner with a collection device. The presence of allergens can be detected and quantified with an ELISA test, even if this method might not provide accurate measurement of inhaled allergens. Successful protocols for allergen avoidance are multifaceted (29). Not surprisingly, there is a high degree of variability between studies on avoidance.

1. Physical barriers should be recommended?

Frequent vacuum cleaning and acaricides have been proved to be not sufficiently effective in reducing HDM exposure, while specific *physical barriers*, in particular pillow and mattress encasing, have been demonstrated to be more useful at this purpose (strength of evidence B). The exchange of humidity without transfer of allergens is the most important property of bed encasing, providing a barrier effect between the human body and HDM in the mattress.

Different special covers are available on the market; those made of plastic, made of permeable synthetic fibers, non-woven synthetics, and finely woven with variable pore size (<10 µm block dust mite allergens; 6 µm also block mites and cat allergens) represent special covers for HDM avoidance (37). In a prospective study, Halken et al. recruited 60 asthmatic children sensitized to HDM who received pillow and mattress encasings or sham encasings (38). After 12 months of treatment, they found that HDM allergen levels were decreased and that the use of inhaled steroids was lower than before treatment (38). Other trials did not show clinical benefit in asthmatic patients by using HDM covers alone (39, 40). Therefore, the use of physical barriers, such as pillow and mattress encasing, should be recommended to decrease exposure to mite allergens, if possible as part of a comprehensive avoidance plan.

2. Is it useful to remove carpets, upholstered furniture, and drapes from the house?

Efforts could be made to *restrict the presence of carpets*, upholstered furniture, and drapes in the environment of the dust mite allergic patients, in particular in the rooms where the patient spends the greatest amount of time, first of all in the bedroom. There is a lack of evidence to support this recommendation alone, but these interventions may be useful as a part of a comprehensive intervention plan.

3. Should patient be advised to control humidity in their house?

Humidifier use should be avoided, while *dehumidifiers* can be used, although these do not generally filter the air as air conditioners do (strength of evidence B).

4. And what to say about aggressive cleaning?

*Dry heat and steam treatments* are two possible interventions that can eliminate HDM and reduce exposure to mite allergens. Washing sheets, pillowcases, mattress pads, and blankets weekly effectively reduces mite counts (41). Some useful advices to reduce HDM levels can be to wash the bedding weekly (strength of evidence B) and using a heated drier, in fact hot water (>130°F) is able to kill mites while cold water may reduce HDM concentrations by 90% (41).

Among the so called *acaricides*, benzil benzoate and tannic acid are the most known; chemicals products showed to have modest effects on reducing mite allergen (strength of evidence B), therefore their use is not recommended.

5. Which are the benefits of combined avoidance interventions?

Several controlled trials have successfully documented a decrease in mite allergen for 6 months or more following combined avoidance interventions (39, 42–47).

Platts-Mills et al. showed a significant improvement in patients with asthma and sensitized to HDM after a long-term dust mite avoidance (48). In this trial were enrolled nine patients who used a hospital room as their bedroom for 12 months and their clinical symptoms and medication use were evaluated at the end of that period: two participants stopped to use all asthma medication and five participants no longer needed inhaled steroids. Symptoms and peak flows improved and five patients showed an eightfold increase in their bronchial provocation tests (48).

A recent meta-analysis was performed to evaluate the effectiveness of allergen avoidance in the prevention of allergic symptoms in previously sensitized patients and newborns that have the potential to develop allergies (49). A total of 14 RCTs were identified among all the articles published from January 1980 to December 2012 about allergen avoidance, of which six RCTs were of previously sensitized patients. The examined allergen exposure reduction was mainly obtained through combined intervention to reduce indoor allergens (removal of pets and molds, air filtration systems and vacuum cleaners, special mattress and pillow covers, cockroach killing). In some trials, allergen counts (i.e., cat dander, mite) were recorded, but their concentration at sampled sites did not always correspond to participant’s exposure. Furthermore, even little amount of allergen can lead to bronchial symptoms (50); this was the reason because authors did not considered the absolute allergen levels to evaluate the efficacy of allergen avoidance measures. This meta-analysis demonstrated that exposure reduction to known allergen sources did not improve lung function (FEV1, PEF) in previously sensitized patients (49).

A study on asthmatic adolescents with exclusive sensitization to mites, demonstrated that bed encasing are effective in reducing bronchial hyperreactivity if compared with placebo and acaricides (benzyl benzoated) (45).

Twenty general practices in two different English cities performed a RCT of 335 children aged 6–16 years suffering from allergic asthma and/or rhinitis to compare the effectiveness of allergen-specific interventions vs usual care (51). Specific allergen avoidance strategies (for tree and grass pollens, pets, HDM, or molds) were provided according to allergy history and SPT. After 1 year, patients receiving specific allergy intervention showed fewer nasal symptoms and a higher QoL index, while no significant changes were observed in bronchial symptoms, health-care utilization, or number of days with impaired daily activities (51).

In conclusion, a comprehensive approach to avoid exposure to HDM [including education, encasings, removing carpets and other mites reservoirs, upholstered furniture, drapes, keeping the humidity below 50%, and vacuuming with a high-efficiency particulate air (HEPA) filter every week] is thought to offer the greatest benefit in reducing mite exposure (strength of evidence A).

6. Which are the new perspectives in the future of HDM avoidance?

Recently, new systems to avoid HDM exposure during night have been investigated; *laminar airflow systems* connected with special filters seemed particularly effective in severe, uncontrolled asthma (52). A German multicenter study included 30 patients among children and adult asthmatic patients with uncontrolled moderate-to-severe disease who were treated with an add-on temperature-controlled laminar airflow (TLA) (53). Data from 4 to 12 months of TLA use showed that the addition of TLA to the patients’ regular medication significantly reduced the number of exacerbations and the utilization of hospital resources and improved asthma symptoms as confirmed by a significant increase of the Asthma Control Test index (53).

The GINA guidelines acknowledge that measures should be implemented wherever possible to prevent the development of asthma and asthma symptoms and exacerbations. However, considering that mite allergens may trigger asthma symptoms, the GINA guidelines conclude that no single avoidance measure is likely to reduce exposure to mite allergens, but also that an integrated approach to avoidance cannot be widely recommended (54, 55).

The evidence-based guidelines for asthma management revised for the National Asthma Education and Prevention Program in 2007 recommends allergen avoidance as part of the management of asthma in patients with known allergen sensitivity (56), while in contrast, a recent meta-analysis concluded that dust mite avoidance is “of no use” in the treatment of asthma.

It is important to note that over half of the reported trials of dust mite avoidance have failed because the proposed measures did not reduce allergen exposure for a sufficient period of time (47, 57). A sustained intervention for at least three to 6 months was necessary to demonstrate clinical benefit. Thus, it should be suggested not only to adopt appropriate avoidance measures, but also to effectively sustain these interventions over time; patients should be advised that symptoms are expected to improve gradually (30).

## Pets

Cats’ and dogs’ allergens are ubiquitous and have been found also in places where pets are not present: a study performed by Bollinger et al. showed that Fel d 1 was detectable in the dust samples from 38/40 homes without cats with a median concentration of 258 ng/g (58). Also clothing are a relevant reservoir of cat allergens: in schools a significant difference in the mean concentrations of cat allergen in the air was found between classes with many and few cat owners (59). In the same study, cat-allergic children who attended classes with more than 18% of schoolmates with a cat at home showed a ninefold increased risk of exacerbation of asthmatic symptoms after the beginning of school than those attending classes with few cat owners (59). In sensitized patients, the exposure to pets is related to an impairment in the lung function. In the Asthma Control Evaluation study which enrolled 546 inner-city adolescents, elevated specific IgE to cat were linked with higher FE_NO_ concentrations, poor lung function, and higher eosinophilia and with an increased risk of exacerbations (60). In another study enrolling 374 adolescents, FE_NO_ levels in pediatric patients with allergic sensitization to cat resulted statistically significant higher in patients with a cat or other furry animals at home than in patients without pets in their indoor environment (61).

### Which Avoidance Measures Should Be Recommended to Pets-Sensitized Children with Asthma?

The advice to families of asthmatic children sensitized to furry animals (cat and dog) is to decrease overall exposure.

1. Should patients remove pets from their homes?

The most effective measure recommended by the AAAAI and the ACAAI is to *remove the pet from the home* (strength of evidence A) (62).

Otherwise, since most families are recalcitrant in removing the animals for affective reasons, specific measures to reducing allergen exposure with the pet still living in the home have been assessed and reviewed (strength of evidence C) (8, 30, 62). Despite of this, parent should be advised that a pet in the house spread a so large concentration of allergens that the clinical benefit and effectiveness of the proposed measures is yet to be proven (62).

2. Which are the benefits of keeping the pet (at least) outdoor?

This measure is effective, but even when cats are removed from the house, allergens persist for many weeks. Wood et al. analyzed the consequence of cat removal in household-dust samples, and they found that allergen concentration begin to decrease several weeks after cat removal, achieving levels similar to control houses without cats into 24 weeks (63). These data may explain the exacerbation of symptoms experienced by subjects allergic to cats after moving into houses which had previously hosted cats (30).

3. Should we recommend the use of air filters?

*Air filtration* reduces indoor levels of airborne allergens. In particular *HEPA air cleaners* are effective in reducing dog and cat allergen concentrations (strength of evidence B), while duct cleaning was not found to be effective (strength of evidence D) (62). Van der Heide et al. conducted a crossover study on 20 asthmatic children sensitized to cat and/or dog and with a pet in the home, who significantly improved their lung function 3 months after the placement of air cleaners in living rooms and bedrooms (64). Systematic reviews on the benefit of air filters and air cleaners suggested that allergic patients should choose one of the following options: portable room air cleaners with HEPA filters, especially during sleeping, or in case of home with heating, ventilation, air-conditioning systems a regular maintenance schedules and the use of HEPA filters (65, 66).

4. Which are the advantages of physical barriers?

*Woven microfiber bed encasings* can block Fel d 1 passage if they are made with pore size lower than 6 µm and may be useful after removing the pet from the home. Meanwhile, the benefit of this measure is still unclear (strength of evidence C) (62). In contrast, non-woven microfiber encasings act as reservoir of allergen and are unsuitable for allergen avoidance (strength of evidence C) (62).

5. Should patients use specific cleaning approaches in their houses?

*Chemical treatments* (e.g., tannic acid) act modifying the allergen structure. Tannic acid is a protein-denaturing agent and its effect on pets’ allergen reduction has been longtime investigated. Despite it has shown a capability to denature Fel d 1 and a subsequent 80% reduction in allergen if applied on furniture or carpets, no evidence of improvement in respiratory health was demonstrated (strength of evidence C) (67). Solution of sodium hypochlorite at 0.05% may be effective in inactivating indoor allergens and reducing allergen exposure. Despite of this, its frequent domestic use (>4 times/week) can be irritant for the respiratory system, increasing lower respiratory tract symptoms (strength of evidence C) (62, 68).

Regular use of *high-efficiency vacuum cleaners* can reduce indoor Fel d 1 and Can f 1 exposure, although the health effects are uncertain (strength of evidence B) (62). The impact of high-efficiency and standard vacuum-cleaners use on indoor allergens levels (mite, cat, and dog) was analyzed in a controlled trial showing lower allergen levels and clinical improvement in the peak expiratory flow rate, FEV1, and bronchodilator use after a 12-month period in homes cleaned with HEPA filters vacuums compared with vacuums with standard filters (69).

Finally, the use of *dry heat* should not be recommended to reduce exposure because Can f 1 and Fel d 1 are very thermostable allergens, in fact allergen concentrations are only partially reduced after 60 min of heating treatment at 140°C, and, respectively, 50 and 70% of allergen remains after treatment (strength of evidence C) (70).

6. Should pets be washed regularly?

The effect of *washing pets* on allergen exposure has been studied (71). Washing pets once or twice a week can reduce the concentration of Fel d 1 or Can f 1 (72) exposure but the effectiveness of this measure in term of better control of asthma symptoms has not been demonstrated (strength of evidence B) (62). Otherwise, washing cats less often does not generate any clinical benefit in patients, since airborne allergen concentration gets back to basal levels within 3 days (71).

7. Do the so-called “hypoallergenic breeds” really exist?

Up to now, no scientific evidence supports the existence of *hypoallergenic cats or dogs* (strength of evidence C) (30). Some breeds of cats (e.g., Siberian cat) have shown lower lever of Fel d 1, but no subsequent lower airborne dispersion of Fel d 1 within the domestic setting has been shown (30). Similarly, as shown in a recent study, no evidence support the classification of certain dog breeds (e.g., Labradoodle, Poodle, Spanish Waterdog, Airedale terrier) as “hypoallergenic” (73). Investigators compared the levels of Can f 1 in hair samples and in the indoor environment of hypoallergenic and non-hypoallergenic breeds and found no differences in Can f 1 levels in the homes of the two groups (73).

The application of the above mentioned measures is essential to control exposure to pets allergens and to improve respiratory health (strength of evidence C) (62).

## Cockroaches

Despite the measures necessary to reduce exposure, the first interventional trials were generally unsuccessful in reducing symptoms (74, 75), maybe because patients could be exposed to high levels of multiple allergens, especially those living in poor conditions, so intervention focused to remove only one allergen can have a poor global impact.

### Which Avoidance Measures Should Be Recommended to Cockroaches-Sensitized Children with Asthma?

Current recommendations to avoid cockroaches allergens include placing multiple baited traps or poisons, eliminating potential food sources, and removing reservoirs of cockroach debris and standing water (76). The allergen settles quickly and does not remain airborne, so air filtration systems are not helpful in reducing cockroach allergen exposure.

## Pests

An American study on inner-city school-age children with asthma examined dust samples from bedrooms to detect the presence of pests allergens and found that the allergen Mus m 1 was present in all the samples (77). In addition, also schools are a source of mice allergen exposure and levels of mice allergen in inner-city schools are often higher than in homes (16). Among dust samples from four different urban schools in the northeastern of US, up to 90% of them contained significant levels of mouse allergens and 68% of them had levels of mouse urinary protein greater than 0.5 µg/g (78). High exposure is considered as a risk factor for asthma morbidity: sensitization to mice and concomitant high levels of domestic exposure to mouse allergens were found to be associated with more physician visits, emergency department (ED) accesses, and hospitalizations in asthmatic preschool-aged children (strength of evidence B) (79). In addition, infants with household exposure to mice were more prone to develop wheezing during the first 12 months of life and in later years (80).

### Which Avoidance Measures Should Be Recommended to Mice-Sensitized Children with Asthma?

Mouse allergen avoidance needs multiple interventions. Multifaceted approach in the homes over a 5-month period was found to be effective in decreasing significantly mouse allergen concentration (81). Moreover, these measures were found to be effective also to control asthmatic symptoms in sensitized children (82).

The main specific control measures include education of patients, cleaning, to cover residues of food and waste, the use of air filters, to repair holes and cracks in indoor environments, and using low-toxicity pesticides and traps (strength of evidence C) (30, 81). Often also professional exterminate on is needed to reduce mouse allergen levels (strength of evidence A) (76).

### Indoor Molds

Since molds are present in environments, the absolute level of spore contamination can be used to make decisions about costly remediation of indoor environments. However, mold spore or mold-specific allergen levels should be interpreted with caution, as these are rarely quantitative and they are often generated using samples that may not be representative of the entire environmental area of concern.

### Which Avoidance Measures Should Be Recommended to Indoor Mold-Sensitized Children with Asthma?

Today, strategies for elimination are not based on scientific evidences.

Reducing humidity by increasing ventilation, covering cold surfaces such as water pipes with insulation, and increasing the air temperature to reduce surface humidity are inexpensive actions that can help discourage mold expansion (83).

A systematic review of 61 published observational studies concluded that exposure to visible mold was associated with increased risk of wheezing and asthma (84). Furthermore, strong evidence has been provided by a birth cohort study, which found that exposure during infancy to measured mold species, especially *Aspergillus fumigates, Aspergillus ochraceus, Aspergillus unguis*, and *Penicillum*, was associated with subsequent childhood asthma (85). A randomized controlled trial in adults with asthma evaluated the effect of removing indoor mold, applying a fungicide, and installing a fan in the loft. In this study, 164 homes housing and 232 asthmatic patients were randomly assigned to undergo cleaning with detergent and fungicide and installation of an attic fan or to have this intervention performed 1 year later. Patients experienced a reduction in the medication use (41% decreased in the intervention group vs 17% increased among control patients) and an improvement of their breathing symptoms of asthma and rhinitis (52 vs 0% in the intervention and control groups, respectively), but without statistically significant evidence of benefit (86). Another similar randomized trial in asthmatic children also found modest benefit (87).

### Avoidance Measures against Outdoor Allergens

Pollens and molds counts are assessed directly (i.e., grains of pollen or mold spores per cubic meter) over a 24-h period, their daily monitoring during a seasons is an important information to control asthma and allergies. The most relevant airborne allergens may be carried by wind for long distances and in several directions, so the complete allergen avoidance cannot be obtained in the real life.

## Pollens

Pollen outdoor exposure and climate variability may induce asthma attacks or exacerbate symptoms in patients with specific sensitization to pollens. In particular, thunderstorms are considered as severe risk factors in asthma exacerbation because rain washing airborne pollen grains and the derived bioaerosols can represent a trigger factor for asthma exacerbations, explaining the so called “thunderstorm asthma epidemics” (27, 28). In Australian cities, up to a 10-fold increase in ED access due to asthma attacks was recorded within 24 h after a thunderstorm (88). The same phenomena happened in London in 1994 when 40 patients presented with asthma exacerbations within 24 h after a thunderstorm (89). Moreover, pollen may increase sensitivity to other airborne allergens, mainly due to its protease enzymes that increase the permeability of the epithelial membranes, disrupting their transmembrane adhesion proteins (90).

### Which Avoidance Measures Should Be Recommended to Pollen-Sensitized Children with Asthma?

Pollens level is difficult to be controlled in the outdoor environment, so avoidance measures are mainly focused on the allergen control in the domestic setting.

Efforts should be focused to reducing indoor exposure and include closing windows and doors during high counts; using air conditioning and HEPA filters in the car and in the home and bathing after being outdoor during pollen to remove allergens from the hair and the body (Table 1) (8).

#### Should Patients Follow Specific Recommendations When They Get Outside during Pollen Season?

Patients should stay inside during thunderstorms and in the midday and afternoon, when pollen counts are highest. In addition, they should wear glasses or sunglasses and a face mask over the nose and mouth during mowing. If possible vacationing in a different ecosystem during pollen season might be suggested (8).

## Outdoor Molds

Allergic sensitization to *Alternaria* has been identified as a risk factor for severe asthma symptoms and also for epidemic asthma (20, 21). An American study performed by Targonski et al. showed that asthma deaths in Chicago increased of two times when the concentration of *Alternaria* spores were higher than 1,000 spores per cubic meter (22). Dales et al. analyzed the relationship between the number of visits for asthma in the ED of a children’s hospital and concentrations of pollens and fungal spores during a 5-year period (91). The concentration of fungal spores significantly correlated with the number of ED visits. Sears et al. performed a study in a group of New Zealand children followed from birth to 13 years. They demonstrated that allergic sensitization to HDM, cat, dog, and indoor mold were significantly associated with current asthma, while grass sensitivity did not show a similar association (23).

### Which Avoidance Measures Should Be Recommended to Outdoor Molds-Sensitized Children with Asthma?

Even if outdoor pollen or mold spore level is difficult to control, the amount of allergen that gets inside home can be object of quantification. During those period when outdoor counts is high, some useful advices may be to close windows and doors and have frequent baths to remove all allergen residues on hair and body (Table 1).

## Conclusion

Over the recent years, a wide spectrum of interventions has been suggested to reduce the exposure to trigger allergy in sensitized patients suffering from respiratory allergy. Although often intuitive strategies, avoidance interventions for the main allergens are not supported by robust evidence of efficacy and are still subject of controversy. Specific control interventions could be effective as part of a holistic approach, so that allergen avoidance is still widely recommended in the most recent guidelines (54, 55) to reduce the severity of allergic respiratory symptoms in sensitized individuals.

## Author Contributions

FC and EC reviewed literature about the selected topic and collaborated in drafting the manuscript. GR examined and corrected the final version of the manuscript. All the authors read and approved the final draft of the manuscript.

## Conflict of Interest Statement

The authors declare that the research was conducted in the absence of any commercial or financial relationships that could be construed as a potential conflict of interest.
